# Money doesn’t matter! Householders’ intentions to reduce standby power are unaffected by personalised pecuniary feedback

**DOI:** 10.1371/journal.pone.0223727

**Published:** 2019-10-23

**Authors:** Kathryn Buchanan, Riccardo Russo

**Affiliations:** 1 Department of Psychology, University of Essex, Essex, United Kingdom; 2 Department of Brain and Behavioral Sciences, University of Pavia, Pavia, Italy; Middlesex University, UNITED KINGDOM

## Abstract

Many researchers have examined whether giving people feedback about their energy use can lead them to decrease it. However, to date no consensus has been reached about which type of eco-feedback is the most effective. We aim to test the efficacy of different feedback techniques by providing participants with personalised information about the annual monetary costs of their home’s standby power usage (i.e., appliances that consume electricity despite not being actively used). Using a sample of 708 participants we tested the following feedback strategies: advice, disaggregation, loss vs gain framing, social norms, and collective information. We measured the impact of each of these feedback conditions on knowledge and intention to change behaviour, and compared them to a control condition. Using both frequentist and Bayesian analyses, we found that relative to the control condition all the feedback strategies led participants to report significant gains in knowledge. Yet, neither the additional knowledge gains, nor the feedback approach used significantly affected behavioural intentions. Consequently, the results suggest that while a wide range of feedback strategies emphasizing the financial impact of standby power consumption can effectively improve knowledge, this approach alone is insufficient in inciting intentions to change energy consumption behaviours.

## Introduction

The worldwide roll out of smart meters presents new opportunities for providing residents with feedback about their energy consumption. This has sparked debate about whether informing residents about their energy use and the associated costs can lead to energy reduction [1]. Researchers have contributed to this debate through conducting field studies and experiments to assess whether energy feedback can promote energy conservation [2,3]. Meta-analyses suggest a significant effect of feedback [4] with average energy savings of 7% [5] although effect sizes vary considerably. Yet, despite a growing knowledge base surrounding the efficacy of feedback, two criticisms can be made of research in this area. The first concerns the combination of multiple feedback strategies (e.g. goal setting *and* tailored advice) within a single feedback intervention [4]. Clearly this is problematic because it is impossible to determine which strategy is responsible for the treatment effects or to compare the relative efficacy of different feedback strategies. The second criticism concerns insufficient examination of the theoretical mechanisms underlying the efficacy of feedback [6]. Yet, if effective feedback interventions are to be implemented, then it is important to know not only if they work but also how they work. While this observation was originally made in the late 1980s, it still rings true today as the number of empirical studies assessing whether feedback can reduce energy consumption still outnumber those assessing the assertions about how feedback does (or does not) work [5]. Yet, if feedback interventions that can effectively influence energy consumption behaviour are to be implemented, then it is important to know not only if they can work but also to explore why they might work.

Given this, the aims of the present research were two fold. First we aimed to examine one of the assertions underlying the efficacy of feedback—that it improves consumers’ knowledge of their energy consumption thereby empowering them to identify and enact energy conservation behaviours. Second, we aimed to compare the efficacy of several different feedback strategies.

### Why energy feedback might reduce energy use: the knowledge deficit rationale

Energy is a problematic medium as it is abstract, intangible, and invisible—visually and consciously [7]. Consequently, consumers’ understanding of their energy use is often low [8]. Given that the average consumer knows little about their energy consumption how can they reasonably be expected to reduce it? Indeed, feedback strategies such as providing either disaggregated information detailing the costs of different energy and/or advice about how to reduce energy use or feed directly into this rhetoric as both strategies have underlying rationales of helping people overcome specific knowledge deficits.

Feedback is viewed as a learning aid [9] that can help “bridge the environmental literacy gap” (p.1999) and in doing so empower people with the knowledge they need to identify energy conservation opportunities [10]. Indeed, various studies have found that feedback improves knowledge of energy use [2,11]), although it is unclear whether this knowledge translates into behaviour change. For instance [12] found that educational materials alone were not effective in reducing electricity consumption. In the present paper, we examined the extent to which giving feedback would lead to gains in knowledge and whether these knowledge gains would mediate the effect of feedback on behavioural intentions.

### Different feedback strategies

In the following section we provide a brief overview of the theory and evidence behind each feedback technique used in the present study, as well as explaining how this strategy was tested within our own research.

#### Pecuniary feedback

From home energy audits and in-home-displays, feedback is often presented to consumers in terms of monetary costs, which unlike kilowatt hours is a unit that consumers can more readily relate to and understand [13], and report a preference for [14].

It comes as no surprise then that energy saving campaigns tend to target people’s financial motives (e.g. “Save money by saving energy”), which logically follows given that energy bills emerged as a number one concern for UK citizens over and above other household bills including petrol, food and mortgage costs [15]. Indeed, [16] note that utility and government-run energy efficiency and conservation programs have often utilised economic messages to enrol participants in energy efficient program offerings.

Yet, despite the popularity of the pecuniary approach to eco-feedback, [5] found that advice about financial impacts or potential monetary savings from undertaking pro-environmental behaviours predicted an *increase* in energy use. They speculate that this is because the financial benefits from saving energy are too small to incite behaviour change. Indeed, in Study 1 [17] found that participants reported that reducing energy use was “not worth it” when it was presented in pounds and pence rather than kilowatts. However, this finding was not replicated in Study 2, leading [17]) to conclude that providing the costs of energy use actions did not demotivate participants to reduce their energy use. Similarly, other research has found that providing electricity saving tips that highlighted prospective monetary savings did not undermine householder’s intentions to conserve electricity [18]. Given the popularity of pecuniary feedback, and assertions that it is more meaningful to customers than kilowatt hours, we focused our investigation solely on the monetary savings that could be achieved through undertaking energy saving actions. However, to test the possibility that the monetary savings that could be gained were not perceived as worthwhile we assessed participants’ appraisal of the monetary savings that they could achieve.

#### Personalised feedback

Feedback is thought to be more effective when it is personalised or tailored [8] so that it is specific to a person and their circumstances. Ensuring feedback is personally relevant may prevent it from receiving less attention and/or having less impact on behaviours [19]. Indeed, [7]’s feedback model states that the realisation of the relevance of one’s own behaviour is a necessary precursor for pro-environmental behaviour, while [20] propose that personalised feedback is processed centrally (rather than peripherally), leading to a more in-depth consideration of the message. Theoretically then, there is good reason to expect that personalised feedback may be effective, and some research provides empirical support for this expectation. E.g., in their meta-analysis [5] found individualised home auditing was one of the most effective strategies for promoting energy savings, while [2] found that householders that were set energy-reduction goals and exposed to a personalised energy-saving intervention, saved significantly more than householders in a no treatment control group. Given the importance of personalisation, the majority of our feedback conditions involved personalisation as we showed participants costs that were specific to their circumstances.

#### Disaggregated feedback

Disaggregated feedback involves presenting a detailed breakdown of the costs of a home’s energy use. Researchers have theorized that disaggregated feedback may be effective in promoting energy efficiency as linking appliance use to energy consumption allows end-users to deduce which aspects of their lifestyles contribute to their consumption [1,7]. While the theory is logical, a systematic review of the literature in this area found no robust evidence that disaggregated energy feedback is any more effective than aggregate energy feedback [21]. In the present study we tested the efficacy of disaggregation by showing participants a breakdown of the costs as well as the cumulative cost.

#### Combining feedback with advice

Feedback that simply states the cost of energy use may prove ineffective as it relies on end-users to deduce which energy-saving actions they must undertake [1]. Indeed, in line with this reasoning research has found that supplementing feedback with actionable information not only improves end-user’s energy literacy but also reduces energy use [11]. Given these promising results, in one of our feedback conditions we provided advice alongside the personalised feedback. Specifically, we adapted advice from EnergyStar’s website about how to slay energy vampires (i.e., reduce costs associated with stand-by power usage) [22].

#### Feedback framed as gains vs. losses

On the basis of prospect theory [23], researchers have theorized that as people focus on losses more than gains, energy information is best framed as preventing a loss rather than providing a gain [24]. Yet, it seems that only one study has investigated these claims empirically. Specifically, [25] found that presenting a water-heater wrap as a way to avoid losing money rather than as a way to save it increased consumers' willingness to make the investment. In the present study we presented losses as money people were wasting versus the money they could be saving.

#### Norms-based feedback

Social norms are widely considered to be an effective technique in promoting household energy conservation [26]. Indeed they help contextualise feedback by providing a standard of behaviour that people can compare themselves to. Researchers have found that descriptive social norms reduced electricity use, but only for those who were using more than average. In contrast, residents that found out they were using less electricity than similar households increased their consumption–a phenomenon termed the boomerang effect [12]. The boomerang effect can be counteracted by combining descriptive norms with injunctive norms (messages of approval for low consumption and messages of disapproval for high consumption) [12]. However, another study found no statistically significant difference in consumption between those receiving individual feedback versus those receiving individual feedback and social norms, leading researchers to conclude that the impact of social norms may previously have been confounded with that of individual feedback [27]. To investigate further, in some of our feedback conditions we provided descriptive social norms alongside the personalised feedback.

#### Collective feedback

Providing collective feedback may help counteract people’s perceptions that the costs of energy use are small by getting them to consider the wider costs involved. Indeed, energy saving campaigns utilise this tactic. E.g., an advert promoting smart meter adoption in the UK, states that “If we all got a smart meter we could save enough energy to power every home in Aberdeen, Cardiff, and Manchester, for a year!” The underlying argument of such campaigns is that everybody has a responsibility to take action [28] and that collectively our actions can make a difference. Indeed, research shows that people ascribe responsibility for conserving energy both to themselves and also to other consumers, indicating a sense of shared responsibility [29].

There appears to be little research into the efficacy of collective messages, although [30] found that a message targeting social responsibility showed no advantage in reducing energy consumption compared to the control condition. To test the efficacy of collective feedback, in one of our conditions we presented the personalised costs of certain energy use actions along with the annual national costs and a message emphasising the importance of everybody playing their part in reducing unnecessary energy wastage [31].

#### Objectives of the current research and hypotheses

The objectives of the current research were to overcoming the critiques of past research and design a study that enabled us to (a) compare the efficacy of several different feedback strategies and their potential for combined effects and (b) to examine the common theoretical assertion behind the efficacy of feedback–that it increases knowledge.

Accordingly, we designed an experiment where we used an online calculator to give participants feedback about their home’s annual standby power consumption levels and then assessed their intentions to enact energy saving behaviours. Appliances that consume energy when in a standby mode (i.e., despite not being actively used) include televisions, internet routers, and game consoles.

We chose to focus on standby power for three reasons. First, standby power usage constitutes 10% of the average American’s energy bill. Second, we reasoned that, compared to other energy saving measures (e.g. purchasing energy star products), unplugging appliances is a relatively easy action to take. Third, as we derived our feedback using calculations extracted from a US utility company’s (NStar) energy vampire calculator we were able to provide each participant with a personalised estimate of their energy vampire costs [32].

We primarily focused on providing personalised feedback to participants about the annual monetary costs of their home’s standby power but also examined whether additive effects were observable when personalised monetary-based feedback was combined with other feedback strategies, namely: disaggregation, advice, loss vs. gain framing, social norms, and collective information. We compared both basic personalised feedback and the more sophisticated types of feedback that supplemented personalised costs with additional feedback strategies to two control conditions: a neutral control (in which participants were told what standby power is) and a generic control (in which participants were told what standby power is and how much it costs the average American home). We hypothesised that personalised feedback would be more effective than either of our control conditions and that the more sophisticated feedback (i.e. the combined effects of personalised feedback with additional behaviour change strategies) would be more effective than personalised feedback alone (*Hypothesis 1*). Given our focus on pecuniary feedback, we also assessed whether participants perceived the money that could be saved through engaging with the feedback as “worthwhile”. In doing so, we aimed to examine speculations that the monetary savings gained from enacting environmental behaviours may be too small to incite environmental action [5, 17].

To examine whether feedback leads to significant gains in knowledge and whether these knowledge gains mediate the relationship between feedback and intentions to enact energy saving behaviours we measured participants’ knowledge of standby power both before and after they were given feedback about the costs of their standby power. We hypothesised that all the feedback conditions apart from the neutral control condition would benefit from a significant increase in their knowledge of appliances that consume energy when in a standby mode (*Hypothesis 2*). Furthermore, we expected that changes in knowledge would mediate the relationship between feedback and behaviour intentions (*Hypothesis 3*).

## Method

Prior to commencement of this research this project was reviewed by the Faculty of Science and Health Ethics Committee at the University of Essex and granted approval with the following number RR1204. The procedure of the study consisted of the following stages which participants completed in the same session.

Informed consent: The first page of the survey consisted of an information sheet where participants were informed about what their participation would involve, what would happen to their data, and their rights as a participant. Participants were given the opportunity to ask questions they had via email before consenting to proceed with the study.

Pre-feedback questionnaire: Following the completion of the informed consent form, participants completed measures assessing their knowledge of standby power.

Instructions for standby power calculator: The instructions stated that participants would be asked to indicate the number of appliances in their home that were typically off, but still plugged in. To ensure participants had read these instructions we asked them to respond to a multiple-choice question asking them to reiterate what they had been asked to do. Specifically participants were presented with the following text. “Just to double check that you understood thee instructions please answer the following multiple choice question: I am being asked to… (a) indicate the number of appliances in my home (b) indicate the number of appliances in my home that are on at any one time (c) indicate the number of appliances in my home that are typically off but still plugged in. After selecting a response item, participants were then given feedback about whether they had correctly understood the task.

Completion of the standby power calculator: Participants indicated how many of the 22 listed appliances they had in their homes that were typically off but still plugged in.

Feedback: Participants received feedback about the costs of their home’s standby power consumption; how this information was presented depended on the condition to which they had randomly been assigned.

Post-feedback questionnaire: participants completed measures of their behavioural intentions to reduce standby power and knowledge of standby power. Participants also provided feedback on the study (e.g., assessment of monetary savings, evaluation of feedback received).

### Participants

To avoid accruing a biased sample of environmental enthusiasts we recruited participants from Amazon’s Mechanical Turk for the intentionally vague task of providing feedback about some information. A total of 708 adult participants from the USA (Mean age = 33.25; age range = 18–80; SD age = 11.17; 45% female) completed the study in 2014 in return for a nominal fee. Note that in our analysis we supplement frequentist analysis with Bayesian analyses thereby negating the need for a power analysis. Nonetheless, based on a moderately small effect size (Cohen’s f = 0.15), a power analysis suggests a sample size of 684 to is required to reach adequate power of 0.80 in order to detect the effect of condition on behaviour intentions.

### Details of feedback presentation

**Fig 1:**
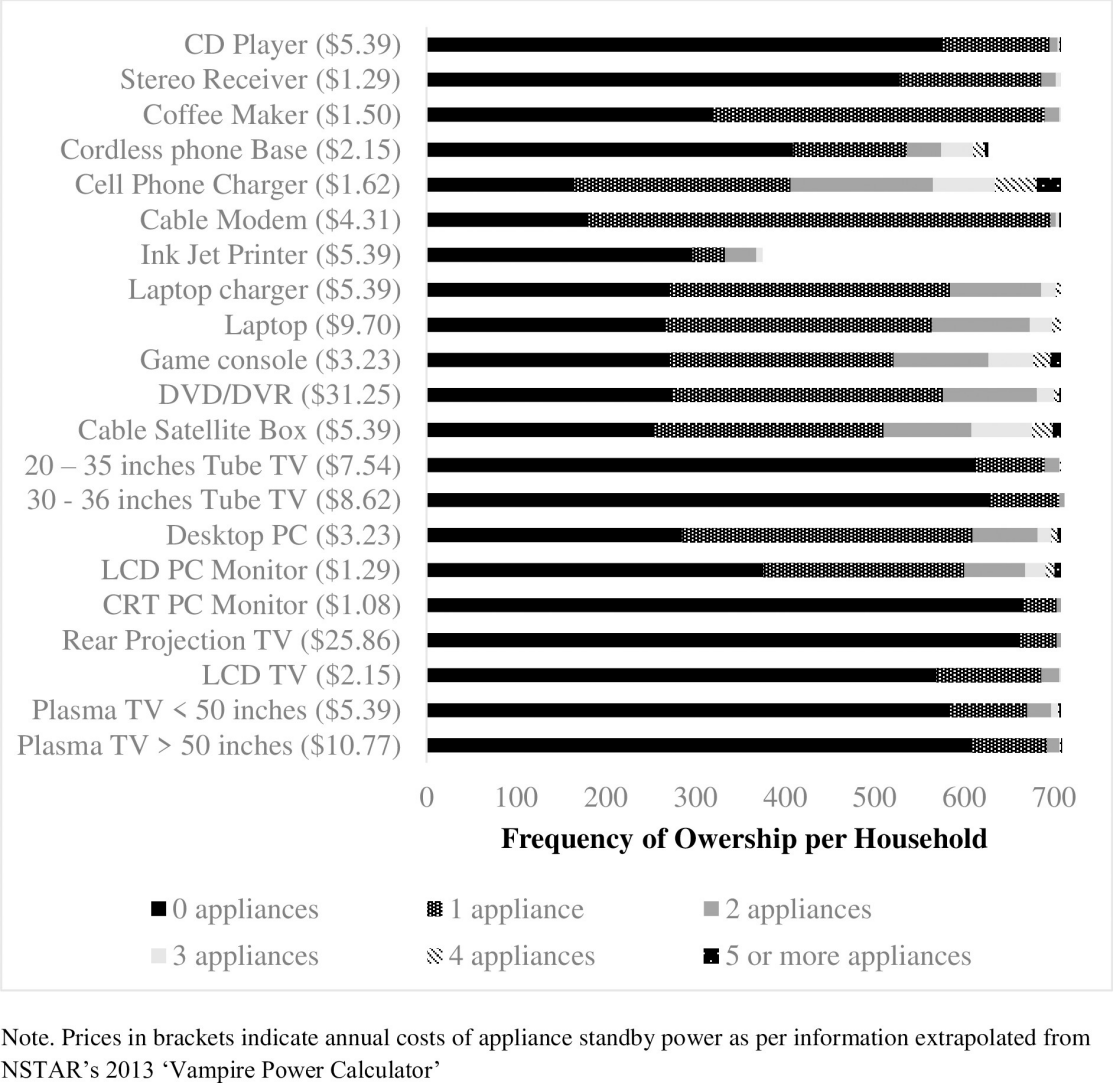
Frequency of ownership of appliances that consume standby power per household.

We presented participants with the annual costs of standby power using the estimates provided by NSTAR’s 2013 ‘Vampire Power Calculator’ (See Fig 1 for details about frequency of ownership of various appliances and annual costs).

The type of feedback participants received depended on the condition to which they were assigned. It is worth noting that rather than using the terminology “standby power” we used the more engaging terminology of “energy vampires” in each of our feedback conditions In the basic feedback condition participants were told what an energy vampire is (“Appliances that consume energy when they are not in use but are still plugged in are called ‘Vampires’) and shown the total monetary cost of their energy vampires per year (hereafter referred to as personalised total—PT). In the two control conditions participants were either shown (i) an explanation what an energy vampire is but no PT (neutral control) or (ii) an explanation about what an energy vampire is and the generic costs of vampires in the United States but no PT (generic control). In the more sophisticated feedback conditions participants were always told what an energy vampire is and the PT as well as some additional information designed to further boost intentions to enact energy saving behaviours. Specifically, this involved combining the PT with either: (a) the disaggregated costs of each energy vampire, (b) advice about how to eliminate energy vampires, (c) a loss or gain frame (loss frame: ☺ “'Bad news!' You are wasting __$ per year by keeping your appliances plugged in when they are not in use!”. Gain frame: ☺ “'Good news!' You could save __$ per year by unplugging your appliances when they are not in use!”) (d) social norms information based on participants input into the ‘Vampire Power Calculator’ (i.e. “You are using less than/more than/are comparable to the average American home”), and (e) collective monetary and environmental costs. Further details about the exact messaging are available in the supplementary materials (S1 Table).

### Measures

Due to the specificity of the constructs assessed we developed the measures listed below. Within each measure we randomized the order in which the items were presented. Unless stated otherwise, participants provided their responses on a 7 point scale ranging from 1 (‘Strongly Disagree’) to 7 (‘Strongly Agree’).

#### Pre-feedback questionnaire (constructs assessed before feedback)

We assessed knowledge of standby power using three items, “I know how much it costs me when I leave appliances in standby mode”, “If I wanted to reduce my energy costs I would know which appliances I should avoid leaving in standby mode”, and “I can see a clear link between my energy use and my energy bills”. Participants gave their responses using a 7-point scale ranging from 1 (‘Strongly Disagree’) to 7 (‘Strongly Agree’). According to the following rule of thumb, whereby >.9 = Excellent, >.8 = Good, >.7 = Acceptable, >.6 = Questionable >.5 = Poor and < .5 = Unacceptable [33], the reliability of the scale was “poor” (α = .55), but this may be expected given that the scale is made up of just 3 items [34]. Further testing showed the items were significantly correlated with each other (*r*‘s ranged from .24 to .34, all *p*’s < .01) that removing items did not improve the reliability of the scale.

#### Post-feedback questionnaire (constructs assessed after feedback)

Please note that items in the measures “behavioural intention to reduce standby power” and “appraisal of monetary savings” use the terminology “energy vampires” to refer to appliances that consume energy when in a standby mode. This terminology was explained to participants (see details of feedback presentation and also S1 Table) when they were presented with feedback. However, we do not use the term elsewhere in the paper at the request of the editorial board. Nonetheless, in the interests of measurement precision we provide the exact wording below.

**Behavioural intentions to reduce standby power:** Participants indicated the likelihood that they would do each of the following items using a scale ranging from 1 (‘Very Unlikely’) to 7 (‘Very Likely’) “unplug each and every 'energy vampire' appliance?”, “Leave ‘energy vampires’ plugged in” (reverse scored), “unplug some 'energy vampire' appliances?”, “regularly check that appliances are unplugged if they are not in use?”, “Quickly forget how much ‘energy vampires’ add to my energy bill” (reverse scored), “Remind others in my home to unplug 'energy vampire appliances?” and “keep a closer eye on appliances that are in ‘standby’ mode”. The scale had excellent reliability (α = 90),

**Knowledge:** As per the pre-feedback questionnaire. The reliability of the scale was questionable (α = .62).

**Appraisal of Monetary Savings:** Participants indicated to what extent they agreed/disagreed with the following three items: “The amount of money that I could save by unplugging 'energy vampires' is so small that it is not worth bothering with.” (reverse scored), “The amount of money I could save by unplugging 'energy vampires' is small but every little helps” and, “I could save a considerable amount of $ by unplugging 'energy vampires'”. The reliability of the scale was approaching acceptable (α = .69).

**Positive appraisal of feedback:** Participants indicated to what extent they agreed/disagreed that the information the feedback was “helpful”, “interesting”, “useful”, “educational”, “of little value” (reverse scored), “forgettable” (reverse scored), and “boring” (reverse scored). The scale had good reliability (α = .87).

### Analytic strategy

To test our research questions we employed the following analytic strategy. First, we performed a classical Analysis of Variance (ANOVA) with appropriate post-hoc follow-up tests. Second, we performed the comparable Bayesian form to obtain a Bayes Factor (BF) in order to compare the null hypothesis model vs the alternative model of differences between conditions. To calculate the BF we used JASP in its default settings for the *a priori* distribution of the parameters [35].

The advantage of using BF in addition to classical statistics is that it can be used as an index to quantify the degree of evidence in favour of either the null or the alternative hypothesis [35]. In the present study, values greater than 1 indicate that the propensity to consider the null hypothesis viable is more likely than the alternative, while values smaller than 1 indicate the opposite. BF values comprised between 1/3 and 3 are inconclusive; values comprised between 3 and 10 provide moderate evidence for the null hypothesis (values between 1/3 and 1/10, for the alternative hypothesis). Thus, larger (smaller) values indicate stronger levels of evidence for the null (alternative) hypothesis.

## Results

### Can feedback influence behavioural intentions?

Table 1 shows the descriptive statistics for participants’ scores on “behavioural intentions to vanquish energy vampires”. The mean scores ranged from 4.20 to 4.67 and varied little across the conditions.

**Table 1:** Behavioural intention to vanquish energy vampires scores per condition.

	*N*	Mean	*SD*	*SE*	Lower Bound *CI*	Upper Bound *CI*
Neutral Control	63	4.20	1.21	.16	3.88	4.53
Generic Control	83	4.35	1.23	.14	4.07	4.63
PT^1^	134	4.43	1.35	.11	4.19	4.66
Revised Control^2^	280	4.35	1.28	.07	4.20	4.50
PT + disaggregated	63	4.46	1.16	.16	4.14	4.78
PT + vanquishing advice	52	4.27	1.41	.18	3.91	4.62
PT + gain frame	47	4.32	1.46	.19	3.94	4.69
PT + loss frame	49	4.67	1.33	.19	4.31	5.04
PT + lower than average	51	4.29	1.41	.18	3.93	4.65
PT + comparable to average	42	4.42	1.21	.20	4.03	4.82
PT + higher than average	68	4.25	1.16	.16	3.94	4.56
PT + collective information	56	4.66	1.21	.17	4.32	5.00

^1^ PT = Personalised total.

^2^Revised Control is not an additional condition but is the amalgamation of the neutral control, generic control and personalised total conditions.

To assess whether basic personalised feedback was more effective in promoting intentions to enact energy saving behaviours, we first compared it to the two control conditions. The ANOVA did not yield significant differences indicating that the personalised total condition was, potentially, no more effective in promoting intentions to vanquish energy vampires than either the neutral control or generic control conditions, (*F*(2,277) = 0.66, MSE = 1.66, *NS*). The Bayes Factor (BF) was 10.89, supporting the null model. On the basis of these analyses we created a single control condition comprised of the PT, neutral control and generic control. We compared this larger control condition to each of the other feedback strategies.

The second ANOVA comparing the consolidated control group to all feedback interventions was also not statistically significant (*F*(8, 699) = 0.86, MSE = 1.43, *NS*). The comparable BF was 239.07 in favour of the null model.

To investigate these outcomes further, we evaluated the efficacy of each of the experimental conditions against the revised control condition using 8 pairwise comparisons, and calculated a BF for each paired comparison. To obtain these BF we used Dienes’ online calculator [36] and assumed a half-normal distribution of the *a priori* effect of the feedback intervention (i.e. the half normal distribution had a standard deviation of 1.325 and a mode of zero; thus, given the maximum intention score could not be greater than 7, the maximum difference between the control and each experimental condition was about 2.65; hence values comprised between zero and 1.325 were considered, on a priori basis to be more likely (about 68%) than values greater than 1.325 (about 32%)).

The results are as follows: Control vs. PT + Disaggregated: BF = 4.36. Control vs. PT + Advice: BF = 9.43. Control vs. PT + Gain frame: BF = 7.52. Control vs. PT + Loss frame: BF = 1.02. Control vs. PT + Social Norms (lower than average): BF = 5.29. Control vs. PT + Social Norms (comparable to the average): BF = 4.88. Control vs. PT + Social Norms (higher than the average): BF = 12.04. Control vs. PT + Collective information = 1.01. These BFs indicate support for the null hypotheses i.e., that the scores on behavioural intentions do not significantly differ between the control condition and any of the experimental feedback conditions.

As a final step, we examined the likelihood of the overall efficacy of the feedback intervention by multiplying each of the BF obtained from the 8 pairwise comparisons. Since BFs are essentially likelihoods, their product can provide an overall index, albeit a crude one, to support either the efficacy of the interventions (overall) or of the lack of their efficacy. When the BFs were multiplied we obtained a value of 98,612.75, indicative of a strong empirical support for the null effect of different forms of feedback against the control condition. To summarise, the results did not support our first hypothesis as personalised feedback was not more effective than either the control conditions or any of the other feedback conditions.

#### Were the costs too small to incite intentions to change?

Providing information about the costs of a home’s annual standby usage did not inspire participants to want to reduce it. One explanation for this may be that the costs were too small to inspire intentions to change. The annual costs ranged from $0 to $348.93, (mean: $78.01, *SD* 39.72, see S1 Fig for frequency of costs). However, participants did not appear to perceive these costs as insignificant as indicated by a mean score of 4.90 (*SD* = 1.17) out of a possible 7 on the positive appraisal of monetary savings scale.

#### Was the feedback appraised poorly?

An alternative explanation for our null findings is that participants simply did not appreciate the feedback so therefore were not prepared to act on it. Yet, participants scored an average of 5.44 (*SD* = .94) out of a possible 7 on the positive appraisal of feedback scale, and variously described the feedback as “eye opening”, “informative”, “enlightening” etc.

### Can feedback increase knowledge about energy use and costs?

Table 2 shows the descriptive statistics for participants’ scores on knowledge about their energy use both pre-feedback (i.e., time 1) and post-feedback (i.e., time 2).

**Table 2:** Knowledge of standby power per condition.

Condition	*N*	Time 1 Mean	Time 2 Mean	Time 2 –Time 1 Δ	SE of Δ	Bayes Factor
Neutral Control	63	4.52	4.48	-.04, *NS*	.15	10.52
Generic Control	83	4.39	4.90	+0.51, *p* < .001	.09	7.25e-7
PT^1^	134	4.26	5.40	+1.14, *p* < .001	.08	1.10e-43
PT + disaggregated	63	4.41	5.51	+1.10, *p* < .001	.12	5.13e-18
PT + vanquishing advice	52	4.43	5.48	+1.05, *p* < .001	.15	1.19e-16
PT + gain frame	47	4.23	5.21	+.98, *p* < .001	.14	1.33e-10
PT + loss frame	49	4.83	5.64	+.81, *p* < .001	.13	1.96e-8
PT + lower than average	51	4.39	5.43	+1.04, *p* < .001	.14	6.89e-12
PT + comparable to average	42	4.38	5.35	+0.97, *p* < .001	.12	4.44e-14
PT + higher than average	68	4.32	5.59	+1.27, *p* < .001	.12	6.85e-24
PT + collective information	56	4.62	5.64	+1.02, *p* < .001	.16	9.50e-9

^1^ PT = Personalised total.

To examine whether feedback increased knowledge we ran a mixed ANOVA where time (pre-feedback, post feedback) was the within-subjects factor and condition (type of feedback) was the between subjects factor. There was a significant main effect of time on knowledge (*F*(1, 697) = 445.86, *p* < .01, partial η2 = .39). Time also interacted with condition to predict the extent of this change in knowledge (*F*(10, 697) = 7.506, *p* < .01, partial η2 = .10). Pairwise comparisons showed that, as predicted, this interaction was driven by a significant increase in knowledge in all of the conditions, apart from the neutral control condition (see Table 2). The generic control condition also had less of an increase in knowledge than many of the more sophisticated types of feedback (e.g. PT + disaggregated). The increases in knowledge change for each of the more sophisticated types of feedback were very similar, so it was not possible to pinpoint the most effective feedback strategy for bolstering knowledge.

To obtain the BFs we used Diene’s online calculator [36] and assumed an half-normal distribution of the a priori effect of the feedback intervention (i.e. the half normal had a mode of zero and a standard deviation of 1/2 of the size of the difference between 7 and the mean of Time-2 scores from Table 2 for each experimental condition; given the maximum possible score could not be greater than 7, values comprised between zero and one standard deviation were more likely (about 68%) than values greater than one standard deviation (about 32%)). The results are shown in Table 2. With the exception of the neutral control condition where there was no increment in knowledge, each BF is very small, thus indicating strong support for the alternative hypothesis (in this case, our second hypothesis) that feedback can lead to significant gains in knowledge.

### Do knowledge gains mediate the relationship between feedback and behavioural intentions?

Hayes PROCESS Model 4 was used to examine whether change in knowledge mediated the effects of feedback condition on behavioural intentions. The indirect effect was tested using a bootstrap estimation approach with 5000 iterations, and feedback condition was entered as a multi-categorical independent variable [37]. As the model does not allow for more than 9 categories, and we had 11 different conditions, we ran 2 sets of mediation analyses. The first analysis included the neutral control, generic control, personalised total, PT + disaggregation, PT + advice and PT + collective information. The second analysis included the neutral control, PT + gain frame, PT + loss frame, and the PT + each of the social norms condition. For each of these analyses, a dummy-coded vector compared the neutral control condition to the other feedback conditions. The results are shown in full in the supplementary analysis (S2 and S3 Tables); overall the pattern is as follows.

Compared to the neutral control group, all the other feedback conditions resulted in significantly higher increases in knowledge gains (*b*’s ranged from .52 to 1.42, all *p*’s < .01). Changes to knowledge predicted behavioural intentions to reduce standby power (*b*’s ranged from .35 to .42, all *p*’s < .01). In all but one case, knowledge changes failed to have a significant indirect effect on the impact of feedback on behavioural intentions (*b*’s ranged from -.36 to .01, all *NS*). Consequently, we did not confirm our third hypothesis as these results did not provide sufficient evidence that change in knowledge mediates the association between feedback and behavioural intention.

## Discussion

Weaknesses in the design of feedback-related research have limited the conclusions researchers have been able to draw about which type of feedback is the most effective and the mechanisms via which feedback operates (e.g. gains in knowledge about energy use). Accordingly, we designed an experiment in which we provided participants with feedback about the annual costs of their home’s standby power. We manipulated the way in which we provided this feedback using behaviour-change techniques derived from psychological theories and empirical research. In doing so, we set out to identify the most effective way of providing feedback and to investigate one of the mechanisms theorised to underlie the success of feedback–that it leads to knowledge gains.

Our results were somewhat surprising and we failed to confirm our first hypothesis as personalised feedback was not more effective than non-personalised feedback, nor were the sophisticated feedback techniques more effective than personalised feedback alone. Consequently, it was not possible to identify any one optimum feedback strategy as they were all equally ineffectual! This is not to say that feedback was without any benefit. We found that aside from the neutral control, each of the conditions resulted in significant gains in knowledge about standby power, thus confirming our second hypothesis. Further analysis showed that knowledge gains did not mediate the relationship between feedback and behavioural intentions. Thus, we did not find support for our third hypothesis.

### The failure of feedback: Possible explanations

Research has often yielded significant results that demonstrate the beneficial effects feedback can have in promoting energy conservation [7]. Why then did the present study fail to replicate these findings? Our results show it is not because the feedback was appraised negatively. Neither was it the case that our feedback did not improve participants’ knowledge of energy consumption issues. The most obvious explanation is that our experimental set-up differed too radically from other studies, both in terms of the way in which feedback was administered and the targeted behaviour.

The feedback we administered differed from previous studies in two ways. First, unlike in-home displays which provide continuous and real-time feedback, we provided feedback to participants only once. While, this “one-shot technique” is comparable to other real-world applications of feedback (e.g. home energy reports, online energy saving calculators), reviews of the literature have found that feedback is most likely to be effective when it is given frequently and over a long time period [7]. Second, research has revealed the importance of presenting feedback using evocative imagery, which we did not incorporate into our feedback techniques [20].

The present study targeted an unusual behaviour–encouraging participants to unplug their home’s appliances that consume energy in a standby mode. To the best of our knowledge no previous studies have used feedback to achieve this goal. Perhaps naïvely, we viewed unplugging appliances that consume standby power as a relatively easy energy-conservation action that is available to most households. It may be that householders actually view unplugging frequently-used appliances as cumbersome and impractical. Moreover, appliances may be designed with the assumption that they will operate in standby modes (e.g. satellite boxes may need to be actively on to record pre-scheduled programmes). Such insights may have come more readily to light if we had not used an online survey with overseas (US) participants. Indeed, this is a limitation of the present research as had we instead utilised an ethnographic study and/or conducted semi-structured interviews we would have been able to collect richer behavioural and/or qualitative data rather than relying on self-reported behavioural intentions.

### Pecuniary feedback

We utilized pecuniary feedback in the present research by informing participants of the monetary costs of energy vampires. We did this because (a) money is an easier unit to relate to and understand than kilowatt hours, (b) consumers express a preference for cost-based feedback and (c) many energy-saving campaigns target the monetary savings that could be achieved through undertaking pro-environmental behaviours. In our experiment, we found no evidence that pecuniary feedback can inspire people to undertake energy-saving actions. This was despite a positive perception of the monetary savings that could be obtained through these energy-saving actions. Such findings are not entirely new, but rather are in line with the conclusions drawn from a meta-analysis which found monetary savings information to be ineffective in reducing energy use behaviour [5]. Subsequent research undertaken since the meta-analysis has also cast doubt on the efficacy of pecuniary feedback. For instance,[14] found that although householders preferred cost-framed feedback it did not reduce energy use, and [18] found that although cost-framed energy saving tips positively influenced long-term electricity saving intentions it did not lead to significant behavioural changes.

It seems then that there is a growing evidence base documenting the failure of pecuniary feedback and we join other researchers [17, 18, 38] in cautioning against the use of it in future energy saving campaigns despite its apparent appeal to consumers in terms of comprehension and relevance.

### Improving knowledge does not lead to behaviour changes

While the feedback we provided to participants failed to have any impact on their behavioural intentions it did succeed in bolstering their knowledge of energy vampires. As such it seems that feedback is not without any benefits but that the rationale underlying the predominant explanation often given for why feedback may work is flawed. This is because there are two assumptions inherent in the explanation that feedback fills a knowledge deficit. These are: (1) that people lack knowledge about their energy use and (2) that once equipped with the relevant knowledge people will make ‘better choices’ [39]. While he first part of this assumption is reasonable—people appear to know little about their energy usage and, actually, such knowledge is a necessary perquisite if energy usage is to be changed, the second part of this assumption does not hold up to scrutiny–having knowledge does not necessarily mean that people will act on it. Indeed, studies have found that although people often have knowledge about the healthy diet choices, they do not adopt them [40]. Similarly, energy consumption is not influenced by knowledge alone–to presume this (a) infers that humans consistently act in a logical and rational way in pursuit of energy efficiency and (b) overlooks the influence of other behavioural determinants such as habits, perceived effort, needs and desires. Indeed, social scientists have been vocal in their criticisms of the information deficit approach [41], arguing that their focus on changing individual behaviour through knowledge acquisition fails to challenge wider public and social trends towards energy intensive lifestyles [42] while ultimately placing the responsibility for energy conservation with householders, rather than other more powerful system actors [43]. Critics of the information deficit approach have argued that future work on energy feedback needs to recognise factors aside from energy literacy. For instance, householders could be encouraged to make normative judgements about their existing energy-use practices to deem what is acceptable or wasteful in terms of the energy they require [41]; or feedback could be designed to encourage people to reflect on wider social conventions, habits and routines rather than solely on their energy use [1]. However, future research is required to assess the efficacy of such approaches.

## Conclusion

Feedback about energy consumption has increasingly been hailed as a silver bullet in the fight against growing carbon emissions. As such there has been considerable interest in identifying the most effective means of giving feedback and the pathways via which it operates. Accordingly, we designed an experiment that allowed us to compare the efficacy of several different feedback strategies by providing people with feedback about the annual costs of their home’s stand-by power consumption. We also examined whether providing feedback would improve participants knowledge of their standby power and whether this knowledge would inspire intentions to reduce it. We found that feedback was not without benefit, as every feedback condition apart from the neutral control reported significant increases in knowledge of stand-by power. However, these knowledge gains were not sufficient to inspire behaviour changes. Indeed, regardless of the strategies we used, none of our experimental feedback conditions were significantly more effective than non-personalised feedback in promoting intentions to vanquish energy vampires. This left us unable to identify a single most effective feedback technique. Instead, we can only conclude that pecuniary feedback and knowledge gains alone appear insufficient in the quest of inspiring householders to reduce consumption associated with stand-by power. We advocate that future research test alternative models of feedback not grounded in the knowledge-deficit approach to behaviour change.

## Supporting information

S1 Fig Frequency of annual standby power costs. (TIF)

S1 Table Messaging used for different feedback conditions. (PDF)

S2 Table Mediation analyses (Set 1): Testing if knowledge gains mediate the relationship between feedback and behavioural intentions. (PDF)

S3 Table Mediation analyses (Set 2): Testing if knowledge gains mediate the relationship between feedback and behavioural intentions. (PDF)
