# Supplementary figures and images for: Money doesn’t matter! Householders’ intentions to reduce standby power are unaffected by personalised pecuniary feedback
Source: PLoS One. 2019 Oct 23;14(10):e0223727. doi: 10.1371/journal.pone.0223727 (PMC6808434; doi:10.1371/journal.pone.0223727)

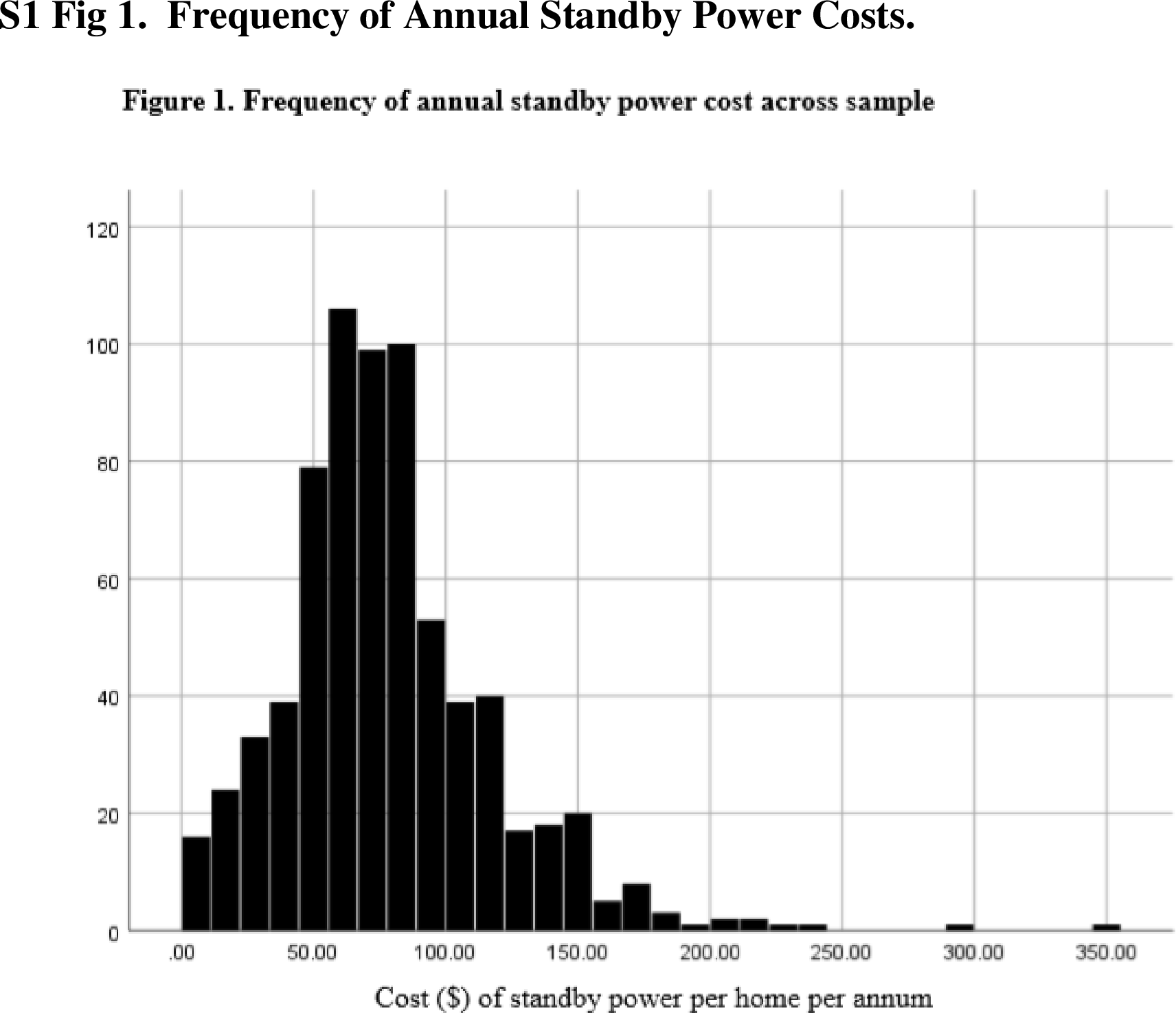

Supplement: S1 Fig — (TIF) [file pone.0223727.s001.tif]
