# Supplementary material for: Money doesn’t matter! Householders’ intentions to reduce standby power are unaffected by personalised pecuniary feedback
Source: PLoS One. 2019 Oct 23;14(10):e0223727. doi: 10.1371/journal.pone.0223727 (PMC6808434; doi:10.1371/journal.pone.0223727)
Supplement: S1 Table — (PDF) [file pone.0223727.s002.pdf]

S1 Table 1. Messaging used for Different Feedback Conditions

Condition	Messaging used
**Control**	"Appliances that consume energy when they are not in use but are still plugged in are called 'Vampires'!"
**Generic total**	"Appliances that consume energy when they are not in use but are still plugged in are called 'Vampires'! In the United States, vampires are adding an additional $100 to each home's energy bills".
**Personalised total (PT)**	"Per year the appliances you leave plugged in but not in use (i.e., in standby mode) are costing you: __ $! Appliances that consume energy when they are not in use but are still plugged in are called 'Vampires'!"
**PT + disaggregated**	As above + a list of cost per each appliance.
**PT + advice**	As above but accompanied by descriptions of the following four actions consumers could take to slay energy vampires:<br>(1) Look for ENERGY STAR products when shopping.<br>(2) Enable ENERGY STAR power management settings on your computer/monitor.<br>(3) Use a power strip as a central "turn off" point.<br>(4) Unplug your chargers.
**Loss frame**	☹ "'Bad news!' You are wasting __$ per year by keeping your appliances plugged in when they are not in use!"
**Gain frame**	☺ "'Good news!' You could save __$ per year by unplugging your appliances when they are not in use!"
**PT + Lower than average**	PT messaging + "Your energy vampire costs are LOWER than the average American home" (shown to participants where costs were less than $48)
**PT + Comparable to average**	PT messaging + "Your energy vampire costs are compare to the average American home" (shown to participants where costs were between $49-$78)
**PT + Higher than average**	PT messaging + "Your energy vampire costs are HIGHER than the average American home" (shown to participants where costs were more than $78)
**PT + collective information**	"But, it's not just you that's plagued by energy vampires. Collectively - a colossal amount of energy is wasted every year. In fact, on a national basis, vampires account for more than 100 billion kilowatt hours of annual U.S. electricity consumption and more than $10 billion in annual energy costs. All this means that it is extremely important that everybody plays their part in reducing unnecessary energy wastage."
