# Supplementary material for: Money doesn’t matter! Householders’ intentions to reduce standby power are unaffected by personalised pecuniary feedback
Source: PLoS One. 2019 Oct 23;14(10):e0223727. doi: 10.1371/journal.pone.0223727 (PMC6808434; doi:10.1371/journal.pone.0223727)
Supplement: S2 Table — (PDF) [file pone.0223727.s003.pdf]

S1 Table 2. Mediation Analyses (Set 1): Testing if Knowledge Gains Mediate the Relationship between Feedback and Behavioural Intentions

		**Behavioral Intention (Y)**				**Change in knowledge (X)**				**Behavioral Intention (Y)**		
		Coeff.	*SE*	*P*		Coeff.	*SE*	*p*		Coeff	*SE*	*p*
**Control vs. Personalized Total (D1)**	*c1*	.22	.20	.25	*a1*	1.12	.15	<.01	*c'1*	-.25	.20	.20
**Control vs. Generic (D2)**	*c2*	.14	.21	.50	*a2*	.52	.17	<.01	*c'2*	-.08	.20	.71
**Control vs. Disaggregated (D3)**	*c3*	.26	.23	.26	*a3*	1.19	.18	<.01	*c'3*	-.25	.23	.28
**Control vs. Advice (D4)**	*c4*	.07	.24	.79	*a4*	1.15	.19	<.01	*c'4*	-.42	.24	.08
**Control vs. Collective (D5**	*c5*	.45	.23	.05	*a5*	1.15	.19	<.01	*c'5*	-.08	.23	.74
**Change in Knowledge (M)**	*b*	-	.-	-		-	-	-	*b*	.42	.06	<.01
**Constant**	*iY*	4.20	.16	<.01	*iM*	-1.09	.12	<.01	*iY*	4.66	.16	<.01
**Model Summary**		$R^2 = .01$				$R^2 = .40$				$R^2 = .12$		
		$F(5, 314) = .90$ $p = .48$				$F(5, 445) = 17.36$, $p < .01$				$F(6, 444) = 9.80$, $p < .01$		
