# Supplementary material for: Money doesn’t matter! Householders’ intentions to reduce standby power are unaffected by personalised pecuniary feedback
Source: PLoS One. 2019 Oct 23;14(10):e0223727. doi: 10.1371/journal.pone.0223727 (PMC6808434; doi:10.1371/journal.pone.0223727)
Supplement: S3 Table — (PDF) [file pone.0223727.s004.pdf]

S1 Table 3. Mediation Analyses (Set 2): Testing if Knowledge Gains Mediate the Relationship between Feedback and Behavioural Intentions

		**Behavioral Intention (Y)**				**Change in knowledge (X)**				**Behavioral Intention (Y)**		
		Coeff.	*SE*	*P*		Coeff.	*SE*	*p*		Coeff	*SE*	*p*
Control vs. Loss frame (D1)	*c1*	.47	.25	.06	*a1*	1.30	.19	<.01	*c'1*	.01	.25	.97
Control vs. Gain frame (D2)	*c2*	.11	.25	.65	*a2*	.92	.19	<.01	*c'2*	-.21	.25	.40
Control vs. Disaggregated (D3)	*c3*	.09	.24	.71	*a3*	1.11	.19	<.01	*c'3*	-.30	.25	.23
Control vs. Advice (D4)	*c4*	.22	.26	.40	*a4*	1.02	.20	<.01	*c'4*	-.14	.26	.59
Control vs. Collective (D5)	*c5*	.05	.23	.82	*a5*	1.18	.17	<.01	*c'5*	-.36	.23	.12
Change in Knowledge (M)	*b*	-	.-	-		-	-	-	*b*	.35	.07	<.01
Constant	*iY*	4.20	.16	<.01	*iM*	-1.09	.13	<.01	*iY*	4.59	.17	<.01
Model Summary		R^2 = .01				R^2 = .18				R^2 = .09		
		$F(5, 445) = .93$ $p = .46$				$F(5, 314) = 13.44$, $p <.01$				$F(6, 313) = 4.97$, $p <.01$		
